# Systems thinking in public health policy development

**DOI:** 10.3389/frhs.2025.1555284

**Published:** 2025-07-14

**Authors:** Alan Silburn

**Affiliations:** School of Medicine, Western Sydney University, Campbelltown, NSW, Australia

**Keywords:** systems thinking, public health policy, modelling methodology, systems approach, policy development

## Abstract

A systems thinking approach is essential for public health policy development, offering a framework to navigate the dynamic complexities inherent in public health issues. This methodology enables policymakers to comprehend the interconnections within public health systems and anticipate the potential consequences of policy implementation. This paper explores the application of systems thinking and modelling methodologies in addressing key public health concerns, such as obesity, infectious diseases, and tobacco use. A review of the literature illustrates how systems thinking informs evidence-based policy creation, improves stakeholder coordination, and mitigates challenges. However, this approach is not without limitations, as unforeseen consequences, financial repercussions, and potential stakeholder biases pose risks. The paper concludes by highlighting the importance of comprehensive, adaptive evaluation mechanisms for ongoing policy refinement, ensuring policies remain effective and relevant in dynamic social, environmental, and political contexts.

## 1 Introduction

A systems thinking approach to public health policy development is essential as it ensures there has been a thorough investigation into the dynamic complexity that characterizes many public health issues. Likewise, the use of a systems thinking approach may define the potential repercussions that can ensue after the policy's implementation. The subsequent sections will reflect how the application of systems thinking and modelling methodology in public health policy development contributes to understanding the dynamic complexities of public health issues and anticipating potential repercussions post-implementation.

Systems thinking is a way of gaining a further understanding of complex situations by identifying the relationships between individual elements of a system and how these elements interact as a dynamic whole (1). In public health, systems thinking is not confined to a single discipline or method. Rather, it incorporates a variety of approaches ranging from systems dynamics modelling and causal loop diagrams to agent-based models and soft systems methodology to explore the linear and nonlinear, qualitative and quantitative, reductionist and holistic dichotomies of population health (2, 3).

### 1.1 Linear vs. nonlinear thinking

Traditional scientific approaches often rely on linear models, where cause-and-effect relationships are direct and proportional an input leads predictably to an output. However, many public health issues arise from complex systems characterized by feedback loops, delays, and emergent properties, which linear models cannot adequately capture. Nonlinear thinking recognizes that small changes in one part of the system can lead to disproportionate and sometimes unpredictable effects elsewhere. For instance, in modelling infectious disease spread or obesity epidemics, nonlinear dynamics such as thresholds, tipping points, and reinforcing feedback loops play a critical (4). Systems thinking embraces nonlinear perspectives, enabling researchers to account for these complexities and better anticipate the unintended consequences of interventions.

### 1.2 Qualitative vs. quantitative approaches

Systems thinking integrates both qualitative and quantitative methods to explore and explain complex phenomena. Quantitative approaches, including system dynamics modelling and agent-based simulations, offer numerical precision and the ability to forecast outcomes under different scenarios. Conversely, qualitative methods such as causal loop diagrams, stakeholder interviews, and participatory mapping provide contextual insights into how system elements interact, the values and beliefs of actors, and the social and political dimensions influencing health outcomes (5). Combining these approaches facilitates a more comprehensive understanding that neither could achieve alone, merging the gap between numerical modelling and lived experience.

### 1.3 Reductionist vs. holistic paradigms

Reductionism involves breaking down complex systems into their constituent parts for isolated study, often assuming that understanding each part will explain the whole. While this approach has driven scientific progress, it falls short in addressing complex public health challenges where interactions and relationships between components generate system-level behaviours. In contrast, a holistic paradigm reinforces the interconnectedness and interdependence of system elements, viewing the system as more than the sum of its parts. Systems thinking prioritizes this holistic view, encouraging researchers and policymakers to consider multiple layers of influence from individual behaviours to societal structures and how they dynamically interact over time (2). This perspective supports more integrative and adaptive policy responses that reflect real-world complexity.

## 2 Background

The Australian Prevention Partnership Centre (6) outlines three defining features of systems thinking relevant to public health. Firstly, it involves a perspective that considers how contributing elements interact to influence the behaviour of the system as a whole. Secondly, systems thinking relies on tools and methods to analyse, describe, and understand complex problems. These include visual representations such as causal loop diagrams or mathematical models that quantify system interactions. Thirdly, it uses terminology that describes the characteristics of a system, such as “reinforcing” or “balancing” feedback loops, which help explain how particular elements may amplify or dampen outcomes across the system (6).

Systems thinking has been consistently applied to public health to identify the interconnections between complex and loosely coupled systems of stakeholders, including governmental entities, non-governmental organizations, and the general population (2). It is frequently employed to examine broad-ranging threats to population health such as obesity, tobacco use, violence, infectious disease, and health inequities, problems often characterised as “wicked” because of their resistance to simple solutions (7). By analysing feedback loops and unintended consequences, systems thinking can help explain how policies designed to target a single risk factor may unintentionally generate adverse outcomes elsewhere in the system (8).

Public health policy, shaped by this perspective, becomes more than just a linear response to epidemiological data. Evidence-based policymaking involves understanding the factors that enhance policy adoption, the components likely to yield impact, and the unintended ripple effects of interventions (9). In this context, systems thinking is frequently promoted as a suitable framework to guide the development, evaluation, and revision of public health policies (3).

However, recent literature raises important critiques of the way systems thinking has been applied in public health. Carey et al. argue that many public health initiatives invoke systems language superficially, without engaging deeply with its underlying concepts (10). They suggest the discourse around systems thinking in health promotion often follows a narrow and instrumental path, with insufficient attention paid to equity, power, and politics (10). Similarly, Peters notes a disconnect between the theoretical promise of systems thinking and its practical application, stating that efforts to integrate systems thinking into health policy are often vague and lack operational clarity (11).

Rutter et al. further highlights the challenges of evaluating complex interventions through a systems lens (4). They argue that many evaluations fall short in explaining how and why change occurs, in part because of methodological inconsistencies and limited use of feedback mechanisms in real-world policy settings (4). In addition, Carey et al. caution that the growing popularity of systems thinking has not always translated into meaningful interdisciplinarity (5). Instead, it can marginalise community-based or indigenous perspectives by privileging technocratic, top-down approaches (5).

Taken together, these critiques do not diminish the potential value of systems thinking but underscore the importance of using it judiciously, with conceptual rigour and reflexivity. It is therefore crucial to recognise that systems thinking is not a panacea. Rather, when applied with care, it can serve as a valuable approach to understanding complex public health problems, evaluating existing policies, and anticipating the broader implications of proposed interventions. Acknowledging both its contributions and its limitations helps to ensure that systems thinking enhances rather than oversimplifies the multifaceted realities of public health policymaking.

## 3 Discussion

The integration of a systems thinking approach into public health policy development represents a paradigm shift that enhances the ability to address complex health challenges. Unlike traditional linear methods, systems thinking acknowledges the multifaceted nature of public health issues, emphasising interconnections, feedback loops, and the interplay of diverse stakeholders. This holistic framework allows policymakers to navigate the intricate relationships between societal, environmental, and economic determinants of health. Public health challenges such as obesity, infectious diseases, and tobacco use serve as prime examples of how systems thinking can inform policymaking by fostering collaboration, improving resource allocation, and enabling dynamic responses to evolving circumstances. However, while systems-informed approaches offer clear benefits, it is equally important to acknowledge the potential for unintended or negative consequences, including issues of equity, financial burden, and institutional inertia.

### 3.1 Obesity

Systems thinking provides a framework for organising the complexity of driving forces attributed to the obesity epidemic and has important implications for policymakers. Gortmaker et al. suggest that various parties, such as governments, international organisations, the private sector, and the general population, need to contribute complementary actions in a coordinated systems approach to reducing obesity through policy (12).

Obesity represents a complex public health issue, where biological, behavioural, social, and environmental determinants interact in dynamic ways. Systems thinking provides a framework to understand these interactions and support policy design. For example, in Chile, the Ley de Etiquetado combined front-of-package labelling, advertising restrictions, and school-based food policy to successfully reduce the consumption of unhealthy foods (13). In comparison, Australia's Health Star Rating System introduced voluntary front-of-pack labelling to guide healthier food choices. While initially promising, the initiative faced criticisms regarding voluntary uptake, industry influence, and its limited effect on consumer behaviour (14). Likewise, Mexico's 2014 soda tax achieved a reduction in sugary drink purchases; however, the long-term effects on obesity prevalence remain unclear due to compensatory behaviours such as increased consumption of other high-calorie items (15).

Further illustrating these challenges, the UK Public Health Responsibility Deal sought to improve food industry practices via voluntary agreements, but critics argue it was co-opted by industry, lacked accountability, and failed to significantly alter consumer behaviours (16). In contrast, Japan's Shokuiku program combines nutrition education with social support and community engagement and has shown promise in preventing obesity through long-term behavioural change and cultural reinforcement of healthy eating habits (17).

Collectively, these international comparisons reveal both the promise and the pitfalls of systems-informed obesity strategies, indicating that success depends on intersectoral coordination, long-term commitment, strong governance, and mechanisms to prevent co-option or unintended substitution effects.

### 3.2 Infectious disease

The study of infectious disease has been the earliest and most important testing ground for systems thinking methods in public health (18). Data from public health surveillance and the systems thinking model are used to determine disease burdens and trends, identify vulnerable or affected people and places, recognise disease clusters, and plan, implement, and evaluate public health interventions and policies (19). During the COVID-19 pandemic, systems-based modelling helped inform effective responses, such as Australia's dynamic border and quarantine policies, which contributed to relatively low early mortality (20). South Korea's pandemic response also exemplifies systems thinking. The country implemented widespread digital contact tracing, rapid diagnostics, and public communication systems, resulting in relatively low death rates without full-scale lockdowns (21). Similarly, Vietnam successfully employed a coordinated whole-of-government approach during COVID-19, combining community engagement, strict quarantine, and contact tracing to limit spread, demonstrating the value of proactive systems integration (22).

However, failures within systems frameworks can have devastating consequences. During the Ebola outbreak in West Africa (2014–2016), containment strategies disrupted local health services and undermined trust in authorities, particularly when communities were excluded from planning (23). In India, the initial COVID-19 lockdown implemented rapidly without broad systems coordination led to widespread displacement of migrant workers and loss of access to essential services (24).

The Netherlands' decentralised COVID-19 response likewise illustrates how fragmented governance can hinder coordination and public compliance, exacerbating the spread of disease despite well-resourced health infrastructure (25). Similarly, Brazil's response to COVID-19 was marked by intergovernmental conflict and inconsistent public messaging, which led to delays in interventions and higher morbidity and mortality (26).

These global examples demonstrate that while systems thinking offers tools for managing infectious diseases, its success relies on governance, equity, transparency, and culturally attuned stakeholder engagement.

### 3.3 Tobacco

Tobacco control has long benefited from systems approaches. Following the 1964 Smoking and Health Report in the US (27), comprehensive strategies like the WHO MPOWER framework guided national efforts. Thailand implemented this multipronged approach—including taxation, advertising bans, and public education—with measurable reductions in smoking prevalence (28). Australia's adoption of plain packaging laws and advertising restrictions further strengthened tobacco control, providing a model of robust systemic regulation (29).

Other countries have implemented similarly comprehensive strategies. For example, Uruguay's stringent tobacco control laws—encompassing graphic health warnings, marketing bans, and smoke-free policies—led to significant declines in smoking (30). Likewise, Turkey successfully reduced tobacco consumption through increased taxation, education campaigns, and enforcement of smoke-free environments (31).

Despite these successes, tobacco control efforts have often faced resistance and adaptation. The tobacco industry continues to undermine regulations through legal challenges, strategic marketing, and lobbying, especially in low- and middle-income countries (32). In Indonesia, lax regulations and industry influence have resulted in high youth smoking rates, and health warnings remain weakly enforced (33).

Further contrasts arise from Canada and Germany. In Canada, plain packaging laws reduced youth smoking initiation and gained broad public support, despite industry opposition (34). Conversely, in Germany, the slow adoption of EU tobacco directives and the persistence of tobacco advertising have contributed to slower declines in smoking rates (35).

E-cigarettes and vaping present newer systemic challenges. In the United States, aggressive marketing led to a surge in youth nicotine use, outpacing regulatory responses (36). New Zealand's initial permissive stance on vaping aimed at harm reduction has since required tighter regulation due to rising youth uptake (37).

Collectively, these examples illustrate how systems-informed tobacco policies must anticipate market adaptation, enforce regulations equitably, and respond to emerging products and behaviours within the broader sociopolitical system.

## 4 Implications for public policy evaluations

The implications drawn from the literature review hold significance for the evaluation of public policies grounded in systems thinking, aligning with established evaluation theories, including process, impact, outcome, and summative evaluation. The adoption of systems thinking in public health policy development has become increasingly recognised for its ability to address the multifaceted nature of health challenges. For example, in Japan, behavioural changes adopted to prevent COVID-19 transmission could serve as a valuable reference for reducing the spread of seasonal influenza (38). Therefore, evaluations must adopt a comprehensive perspective, particularly when assessing the need for policy revision in response to environmental, psychosocial, or political influences impacting the systems model.

First and foremost, the bidirectional interconnections emphasised within the public health system demand evaluation. Evaluators must delve into the collaborative efforts among diverse stakeholders, ranging from governmental entities (e.g., municipal health departments, federal agencies) to non-governmental organizations (e.g., community health centres, advocacy groups) and the general population. The effectiveness of interagency coordination and community engagement becomes pivotal, reflecting the holistic nature of systems thinking-informed policies.

The recognition of unforeseen consequences, despite thorough investigations during policy development, signals a critical aspect for evaluations. Assessments should extend beyond immediate outcomes, incorporating mechanisms for continuous monitoring (e.g., regular surveys to track changes in public attitudes or behaviours related to a policy) and feedback loops (e.g., incorporating community feedback into policy adjustments). This adaptive approach is crucial to identify potential shortcomings and promptly respond to emerging challenges, ensuring the ongoing relevance and effectiveness of policies.

Environmental conditions (e.g., climate change, pollution levels), psychosocial determinants (e.g., social norms, mental health trends), or political developments (e.g., changes in government leadership, shifts in public opinion) can exert significant influence on the systems model underlying public health policies. Evaluators must be attuned to these influences, recognizing that external dynamics may necessitate revisions to the existing policy framework. A vigilant examination of the interconnected relationships between policy elements and the broader socio-political landscape becomes imperative, integrating process, impact, outcome, and summative evaluation approaches. Identifying shifts in environmental conditions, changes in psychosocial determinants, or political developments that impact the efficacy of the systems model can guide evaluators in determining the need for policy revisions.

## 5 Conclusions

The adoption of systems thinking in public health policy development has become increasingly recognized for its ability to address the multifaceted nature of health challenges (39, 40). This is exemplified in the development of comprehensive strategies to combat obesity, infectious diseases, and tobacco use aforementioned. By framing public health systems as dynamic entities characterized by interrelated elements, systems thinking provides policymakers with a robust toolset for understanding and navigating complexity. The successful implementation of public health policies for issues like obesity, infectious disease, and tobacco use demonstrates how this approach enhances stakeholder collaboration, ensures evidence-based decision-making, and promotes sustained health outcomes.

Systems thinking offers a means to identify relationships that might otherwise be overlooked, such as the coordination required among governmental (e.g., public health agencies), non-governmental organizations (e.g., community-based advocacy groups), and the general population. Policies designed with this framework integrate diverse perspectives, enabling more effective interventions. For example, obesity prevention policies benefit from a systems perspective by uniting efforts across sectors, while infectious disease policies leverage data-driven insights to optimise surveillance and intervention strategies. Similarly, tobacco control policies illustrate how systems thinking not only shaped early interventions but also continues to guide adaptive strategies in response to new challenges.

Despite these strengths, the approach is not without challenges. Even the most thorough systems thinking analysis cannot predict all outcomes. Unforeseen consequences, such as industry counterstrategies (e.g., marketing campaigns designed to undermine public health messages) or economic impacts (e.g., increased healthcare costs), underscore the need for vigilance in policy evaluation. Financial biases (e.g., funding priorities that favour certain interventions over others) and political biases (e.g., policies influenced by lobbying efforts) may also influence policy development and implementation, potentially undermining the intended public health benefits. These limitations call for the integration of continuous monitoring and adaptive feedback loops into policy evaluation frameworks.

Evaluations grounded in systems thinking must encompass process, impact, outcome, and summative dimensions to account for external influences such as environmental changes, psychosocial shifts, and political developments. For example, the financial ramifications of policies addressing obesity, infectious diseases, and tobacco use demand scrutiny, as they may place burdens on industries, governments, or individuals. Furthermore, an adaptive evaluation approach ensures that policies evolve in tandem with emerging challenges, maintaining their relevance and efficacy.

In conclusion, systems thinking provides a valuable framework for public health policy development by fostering an in-depth understanding of complex systems and enabling policymakers to anticipate and mitigate challenges. While the methodology offers significant benefits, its success hinges on comprehensive, adaptive evaluation mechanisms that account for dynamic influences and unforeseen outcomes. By committing to continuous learning and iterative improvement, public health stakeholders can harness systems thinking to create policies that address today's challenges while preparing for tomorrow's uncertainties.
